# Improving outcomes with early and intensive metabolic control in patients with type 2 diabetes: a long-term modeling analysis of clinical and cost outcomes in Italy

**DOI:** 10.1007/s40200-024-01553-w

**Published:** 2025-01-29

**Authors:** Pierluca Arietti, Kristina Secnik Boye, Maurizio Guidi, Jonathan Rachman, Marco Orsini Federici, Rosanna Raiola, Arianna Avitabile, William J. Valentine

**Affiliations:** 1 Eli Lilly and Company, Sesto Fiorentino, Italy; 2 Eli Lilly and Company, Indianapolis, IN USA; 3 Eli Lilly and Company, Basingview, Hampshire, UK; 4 Ossian Health Economics and Communications GmbH, Bäumleingasse 20, Basel, 4051 Switzerland

**Keywords:** Type 2 diabetes, Italy, HbA1c, Body mass index, Metabolic control, Modeling study

## Abstract

**Objectives:**

This analysis quantifies the potential long-term clinical and cost benefits of early and intensive metabolic control (EIMC) versus conventional management in patients newly diagnosed with type 2 diabetes in Italy.

**Methods:**

The PRIME T2D Model was used to project clinical and cost outcomes over long-term time horizons for a newly diagnosed cohort of patients receiving EIMC or conventional management. EIMC was associated with a mean glycated hemoglobin reduction of 0.6% from baseline and a mean weight loss of 9.5 kg (8.2%) for a duration of 6 years, before gradually returning to the same levels as the conventional management arm over 6 years. Modifiable risk factors were assumed to progress over time based on published regression functions. Direct and indirect costs associated with diabetes-related complications were accounted in 2021 Euros (EUR), with unit costs and health state utilities derived from published sources. Future costs and clinical benefits were discounted at 3% annually.

**Results:**

For the population diagnosed with type 2 diabetes in 2021 (estimated at 216,417 cases), EIMC was projected to add approximately 33,112 years of life and 55,403 quality-adjusted life years versus conventional management. Cost savings with EIMC were estimated at EUR 494 million, EUR 608 million and EUR 628 million in the incident population at 10- and 20- and 50-year time horizons, respectively.

**Conclusions:**

According to this modeling study, early and intensive metabolic control has the potential to substantially improve clinical outcomes and reduce economic burden compared with conventional management of patients with type 2 diabetes in Italy.

**Supplementary Information:**

The online version contains supplementary material available at 10.1007/s40200-024-01553-w.

## Introduction

Type 2 diabetes is associated with a substantial global economic and clinical burden and, despite recent advances in therapy, remains one of the most significant challenges facing healthcare systems around the world [1]. In Italy, the prevalence of diabetes (type 1 and type 2) in the year 2022 is around 10%, with approximately 4,470,300 cases of diabetes in the total adult population, of which approximately 90% are estimated to be type 2 diabetes [2, 3]. The annual healthcare costs of Italians with diabetes are estimated to be 2.2 times higher than those without the disease, resulting in annual excess costs of approximately EUR 9 billion in 2018 [4]. The majority of this economic burden results from complications associated with the disease, and the direct costs of an individual with diabetes increases linearly with the number of complications [5, 6]. A recent observational study of the Italian Associations of Medical Diabetologists (AMD) reported a prevalence of approximately 33% in microvascular disease as well as over 15% prevalence of any cardiovascular disease in people living with type 2 diabetes in Italy [7].

It is well established that controlling modifiable risk factors, such as blood glucose levels and body weight, can reduce the risk of both micro- and macrovascular complications in patients with type 2 diabetes [8]. Modern therapies, such as glucagon-like peptide-1 receptor agonists (GLP-1 RA) and sodium–glucose co-transporter 2 inhibitors (SGLT-2 inhibitors), have been shown to effectively improve glycemic control, reduce body weight and independently reduce the risk of cardiovascular complications [8]. Such therapies represent important progress in terms of management programs aimed at reducing the progression and burden of diabetes and its resulting complications [8]. The conventional treatment paradigm for type 2 diabetes in the last 20 years has centered on the gradual intensification of therapy as the disease progresses and beta-cell function diminishes [9]. Consequentially, a proportion of the patients are limited to poor glycemic control over the course of treatment, i.e. spending unnecessary time with glycated hemoglobin levels (HbA1c) above recommended targets before therapy is intensified. This repeated and delayed treatment adjustment might further exacerbate the risks as well as costs of diabetes-related complications. Given that previously published studies have indicated that more than 80% of expenditure on type 2 diabetes is associated with the management of diabetes-related complications, there is considerable potential for early, intensive metabolic control (EIMC) to improve outcomes for patients with type 2 diabetes while simultaneously reducing the burden associated with the disease [4]. EIMC can be defined as early treatment, initiated very soon after diagnosis, and intensive management of risk factors such as HbA1c, body weight, blood pressure and serum lipid levels, with specific targets defined for each individual patient. EIMC would involve the use of multiple different class and types of medication across the population to help each individual reach appropriate targets.

Data from recent studies support early, intensive metabolic control interventions at disease onset instead of conventional treatment to reduce risk of complications and emerging economic burden in the long term [4]. Intensive therapy has been associated with significant (and durable) improvements in glycemic control as well as insulin sensitivity and beta-cell function, which, in turn, reduce microvascular and cardiovascular events as well as mortality [10, 11]. The aim of this analysis, therefore, is to quantify the potential long-term clinical and cost benefits associated with an intervention providing EIMC versus conventional management in patients with type 2 diabetes in Italy.

## Methods

### Modelling approach

**Fig. 1:**
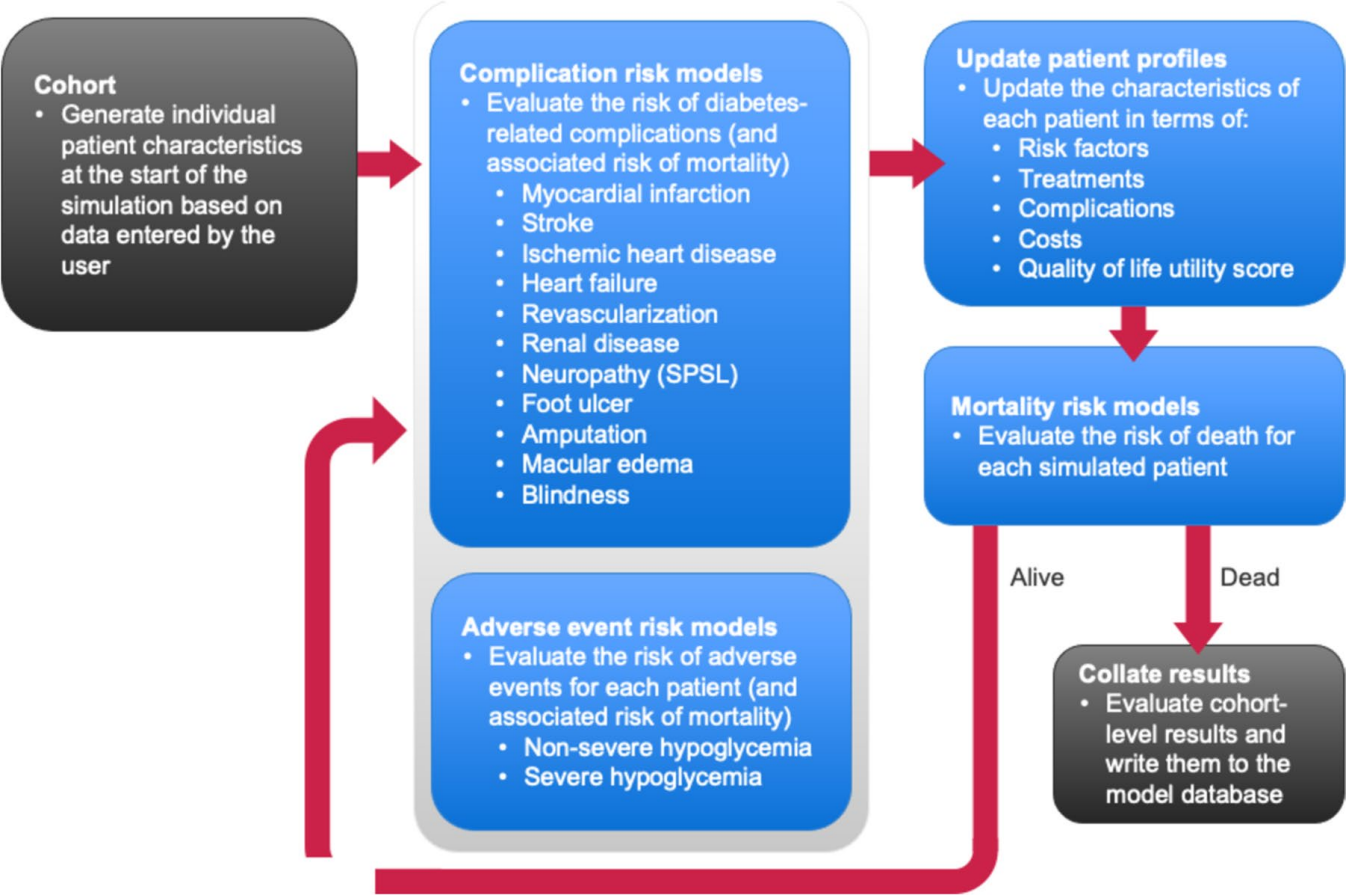
Schematic overview of the PRIME T2D Model used to project long-term outcomes of the analysis

The PRIME Type 2 Diabetes Model (PRIME T2D Model) was used to evaluate direct and indirect costs as well as clinical outcomes (quality-adjusted life expectancy, life expectancy, undiscounted life years) for a cohort undergoing either EIMC or conventional management. The PRIME T2D Model is a product-independent analysis tool to evaluate long-term outcomes for populations with type 2 diabetes and has been validated against real-life clinical data. The model is designed to simulate disease progression, diabetes-related complications, adverse events and mortality as well as to evaluate life expectancy, quality-adjusted life expectancy, direct and indirect costs, along with standard measures of cost-effectiveness [12]. The PRIME T2D Model combines published risk equations (using a weighted model averaging approach for several complications) and Monte Carlo methods to evaluate the risk of mortality and diabetes-related complications based on simulated patient characteristics, risk factors and history of complications [13]. A schematic overview of the model can be found in Fig. 1. For all modeling simulations, 300,000 simulated patients were run through the model in each treatment arms based on the settings described in the *Baseline cohort characteristics and interventions* section below. All analyses were patient-level, first order Monte Carlo simulations with sampling from distributions around cohort characteristics at baseline (to generate simulated patients) and treatment effects (to quantify the effects of EIMC and conventional management on HbA1c and body weight). No probabilistic sensitivity analysis was performed as uncertainty around the magnitude of EIMC benefits was explored directly in scenario analysis (see below).

### Baseline cohort characteristics and interventions

Baseline cohort data characteristics were collected from the Verona Newly Diagnosed Type 2 Diabetes Study, which analyzed an Italian population newly diagnosed with type 2 diabetes in 2021 (*n* = 216,416) [14]. This cohort was chosen in line with the aims of the analysis (investigation of early and intensive metabolic control requires a newly diagnosed cohort) and was assumed to be representative of a newly diagnosed population with type 2 diabetes in Italy. In the modeling analysis, the mean baseline age of the population was 60 (standard deviation: 10.4) years, mean baseline HbA1c was 6.6 (0.8) % and mean BMI was 29.3 (4.7) kg/m^2^. Mean duration of disease of the selected population was 0.3 (0.13) years. Long-term outcomes associated with two interventions were evaluated in this population. In the EIMC group, mean HbA1c was reduced to 6.0% with weight loss of 9.5 kg (equivalent to 8.2%) in the first year after diagnosis. This HbA1c target can be considered achievable given the low mean baseline HbA1c level (6.6%) of the population and HbA1c reductions to near-normal levels (HbA1c 5.7%) in recently reported SURPASS trials [15]. The weight loss target is based on gold standard weight loss in trials of type 2 diabetes interventions (approximating weight loss target with tirzepatide 10 mg in the SURPASS-2 trial over 40 weeks) [16]. Benefits persisted for 6 years, before risk factors gradually returned to the same levels as the conventional arm over years 7–12 (i.e. HbA1c of 8.6%). A duration of 6 years was selected to correspond to initial treatment and one treatment intensification step (e.g. metformin for 3 years and then metformin plus GLP-1 receptor agonist for 3 years) [17, 18].

For conventional metabolic control, the assumption was made that mean HbA1c would gradually increase over time for the population, reaching 8.6% after 6 years of therapy, which is consistent with patients intensifying dual oral therapy in the AMD Annals population [19]. By year 7 of treatment in the conventional arm in the modeling simulation, it was assumed most patients had transitioned to insulin therapy and HbA1c remained constant at 8.6% thereafter. All other modifiable risk factors including blood pressure, serum lipid levels and body weight followed a natural progression over time based on equations from the UK Prospective Diabetes Study Outcomes Model 2 (UKPDS OM2) [20].

### Cost and utility inputs

The direct healthcare costs associated with complications were taken from published sources and expressed in 2021 Euros (EUR) [21]. Indirect costs associated with lost workplace productivity were based on published days off work estimated for diabetes-related complications, Italian salary information and employment rates for simulated patients aged 67 years and younger [22–25]. Quality-adjusted life expectancy was estimated based on published utility scores and combined using an additive approach [26–32]. Future costs and clinical benefits were discounted at 3% annually in line with published recommendations and in line with standard practice to convert future cost and clinical benefits to current values [33]. UKPDS OM2 risk equations were used to evaluate mortality associated with diabetes-related complications and 2019 Italian life tables were used to evaluate the risk of mortality from other causes [34]. Outcomes were evaluated at 10-year, 20-year and 50-year time horizons.

### Supporting scenarios

As supporting scenarios, clinical and economic outcomes were estimated for EIMC across 10-, 20- and 50-year time horizons. The modeling approach followed the same one described in the base case analysis, using equivalent inputs and assumptions except for HbA1c levels and weight loss targets. The analysis estimated the outcomes based on HbA1c levels between 5.7% and 6.9% (with incremental steps of 0.3%) and weight loss between 1.5 and 9.5 kg (with incremental steps of 2.0 kg).

## Results

### Clinical outcomes

**Fig. 2:**
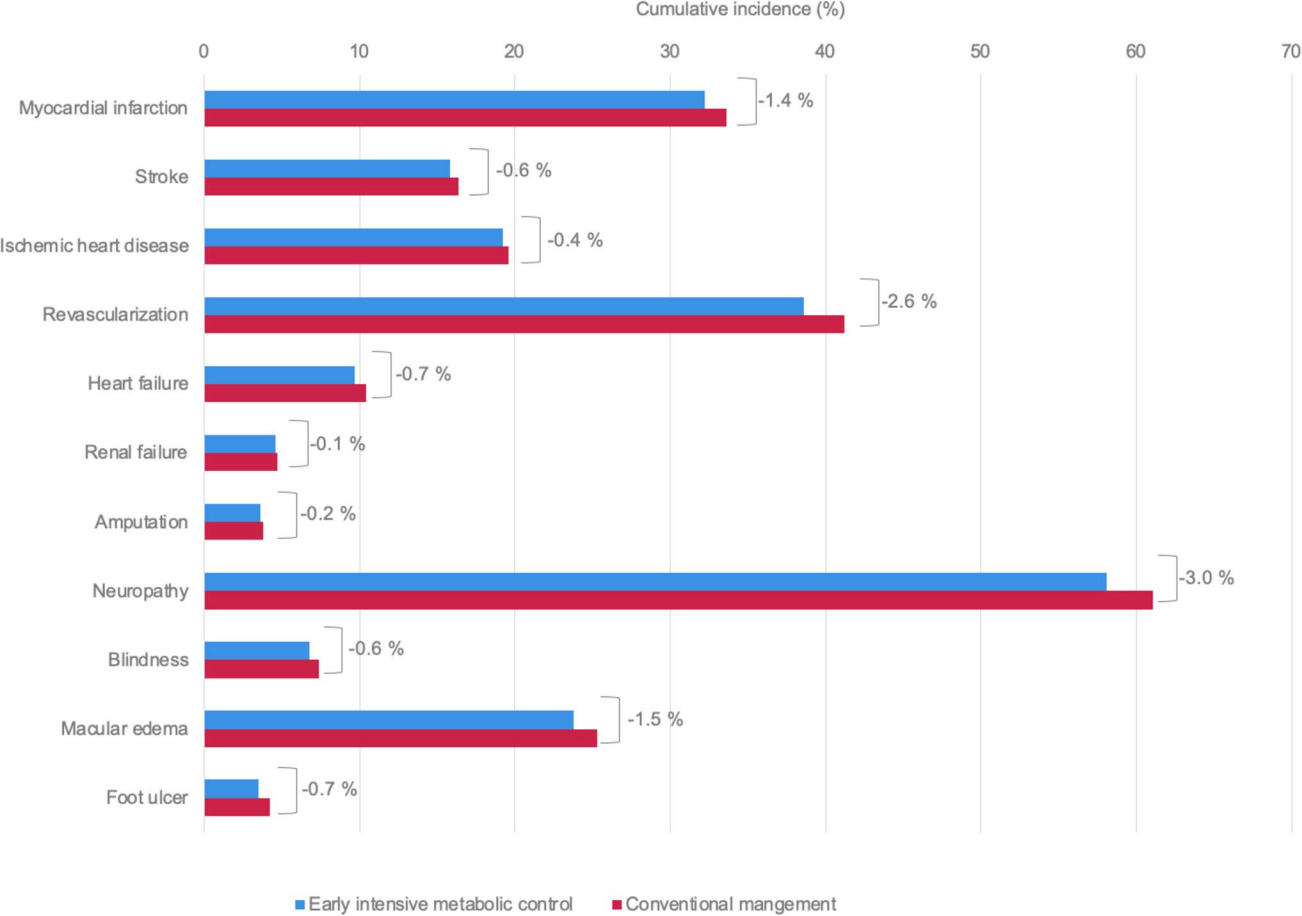
Cumulative incidence of complications for EIMC (gradual HbA1c return) versus conventional management over patients’ lifetimes

EIMC was associated with substantial improvements in long-term clinical outcomes versus conventional management (Table 1; Fig. 2). Reduced rates of diabetes-related complications with EIMC led to an additional 0.15 discounted life years and 0.26 QALYs per patient over 50 years (Table 1). The majority of these benefits were accrued relatively early in the simulation, with 57% of the quality-adjusted life expectancy benefit evident after 10 years (+ 0.15 QALYs) and 88% of the total benefit evident at a 20-year time horizon (0.23 QALYs, Tables 2 and 3). For the incident population (i.e. patients diagnosed with type 2 diabetes in Italy in 2021), these benefits would translate to adding approximately 33,112 life years (discounted value) and around 55,403 QALYs to the population over their lifetime (Table 1).

**Table 1:** Potential clinical benefits and cost savings of EIMC versus conventional metabolic control in Italy over the next 50 years for patients newly diagnosed with type 2 diabetes in 2021

		Undiscounted life expectancy (years)	Discounted life expectancy (years)	Quality-adjusted life expectancy (years)	Direct cost (EUR)	Total costs (EUR)
Patient level	EIMC	17.66	12.99	10.06	50,644	55,767
	Standard care	17.42	12.84	9.80	53,008	58,669
	**Difference**	**+ 0.24**	**+ 0.15**	**+ 0.26**	**−2**,**364**	**−2**,**902**
Population level	EIMC	3,821,931	2,811,262	2,177,159	10.960 million	12.069 million
	Standard care	3,769,991	2,778,799	2,120,890	11.472 million	12.697 million
	**Difference**	**+ 52**,**373**	**+ 33**,**112**	**+ 55**,**403**	**−511.6 million**	**−628.0 million**

Population size is based on data of newly diagnosed patients with type 2 diabetes in 2021

**Table 2:** Potential clinical benefits and cost savings of EIMC versus conventional metabolic control in Italy over the next 20 years for patients newly diagnosed with type 2 diabetes in 2021

		Undiscounted life expectancy (years)	Discounted life expectancy (years)	Quality-adjusted life expectancy (years)	Direct cost (EUR)	Total costs (EUR)
Patient level	EIMC	14.29	11.44	8.91	41,716	46,545
	Standard care	14.12	11.32	8.68	44,001	49,356
	**Difference**	**+ 0.17**	**+ 0.12**	**+ 0.23**	**−2**,**285**	**−2**,**811**
Population level	EIMC	3,092,604	2,475,815	1,928,279	9.028 million	10.073 million
	Standard care	3,055,813	2,449,845	1,878,503	9.523 million	10.681 million
	**Difference**	**+ 36**,**791**	**+ 25**,**970**	**+ 49**,**776**	**−494.5 million**	**−608.3 million**

Population size is based on data of newly diagnosed patients with type 2 diabetes in 2021

**Table 3:** Potential clinical benefits and cost savings of EIMC versus conventional metabolic control in Italy over the next 10 years for patients newly diagnosed with type 2 diabetes in 2021

		Undiscounted life expectancy (years)	Discounted life expectancy (years)	Quality-adjusted life expectancy (years)	Direct cost (EUR)	Total costs (EUR)
Patient level	EIMC	8.81	7.80	6.14	25,075	28,344
	Standard care	8.76	7.76	5.99	26,943	30,625
	**Difference**	**+ 0.05**	**+ 0.04**	**+ 0.15**	**−1**,**868**	**−2**,**281**
Population level	EIMC	1,906,637	1,688,056	1,328,803	5.427 million	6.134 million
	Standard care	1,895,816	1,679,399	1,296,340	5.831 million	6.628 million
	**Difference**	**+ 10**,**821**	**+ 8**,**657**	**+ 32**,**463**	**−404.3 million**	**−493.6 million**

Population size is based on data of newly diagnosed patients with type 2 diabetes in 2021

### Economic outcomes

EIMC effectively reduced costs of type 2 diabetes management (excluding pharmacy costs) by approximately EUR 2,902 per patient over a lifetime compared with conventional metabolic control (Table 1). The majority of cost savings over a lifetime were due to a reduction in direct healthcare costs associated with complications avoided (81% of the total saving) with the remainder due to lost productivity avoided. The greatest direct cost savings were associated with the prevention of cardiovascular diseases (EUR 881 per patient), followed by neuropathy and diabetic foot complications (EUR 538 per patient), lost workplace productivity (EUR 538 per patient), renal disease (EUR 490 per patient) and ocular complications (EUR 395 per patient) compared with conventional metabolic control. For the population diagnosed with type 2 diabetes in 2021, EIMC could reduce the total costs associated with diabetes-related complications over a lifetime by as much as EUR 628 million versus conventional metabolic control. The savings on a population level at 10- and 20-years were estimated at EUR 493.6 million and EUR 608.3 million, respectively (Tables 2 and 3). The majority of cost savings were observed in the first 10 years of the simulation, where 79% of total lifetime cost savings were accrued.

### Incident population

**Fig. 3:**
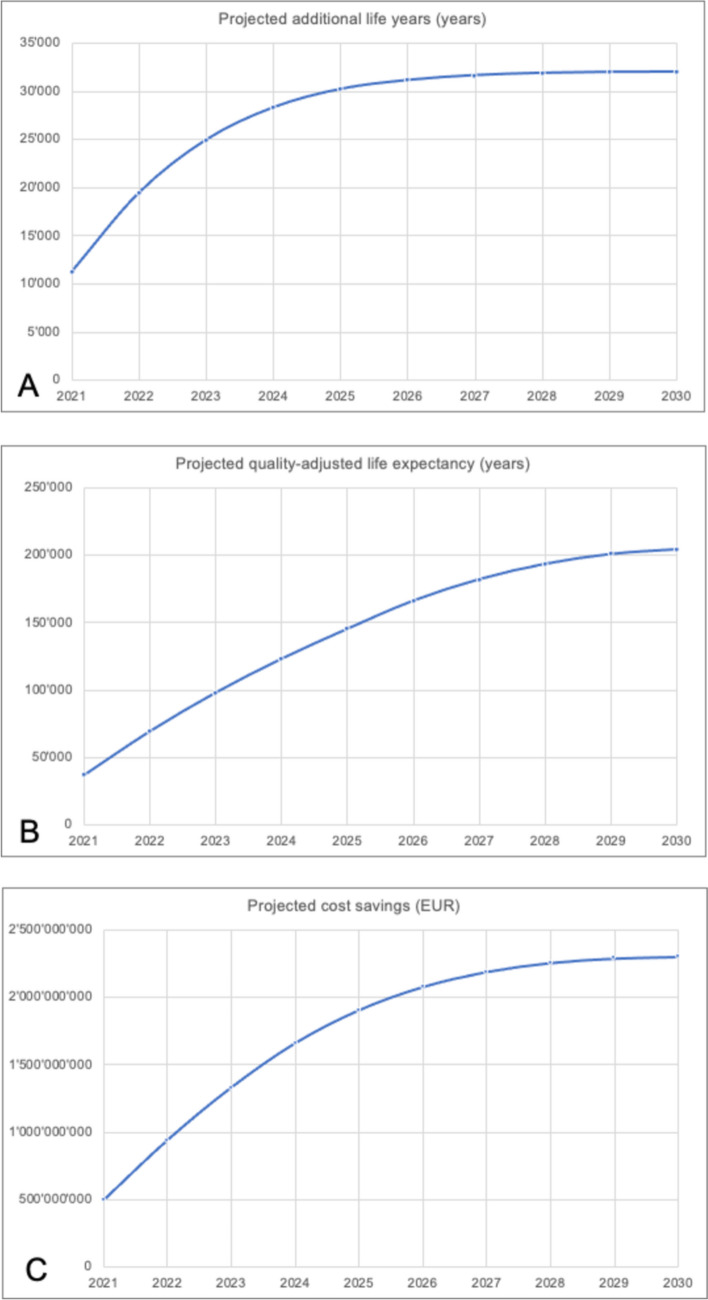
Potential cumulative clinical benefits and costs savings of EIMC versus conventional metabolic control for incident type 2 diabetes patients over the next 10 years in Italy. The graphs show the cumulative benefits over time for the incident populations, diagnosed with type 2 diabetes, in years 2021 through to 2030. Panel a shows the cumulative life years gained for EIMC versus conventional management. Panel b shows the cumulative quality-adjusted life years gained for EIMC versus conventional management. Panel c shows the cumulative cost savings for EIMC versus conventional management

Conservatively assuming that the incidence of type 2 diabetes remains constant in Italy over the next 10 years, EIMC was estimated to add approximately 32,009 life years to those diagnosed with the disease in that time frame compared with conventional metabolic control. EIMC could bring an additional 204,143 QALYs and save an estimated EUR 2.3 billion in the next 10 years compared with conventional metabolic management (Fig. 3). These estimates reflect the total benefits accrued in the incident populations each year from 2021 to 2030 and could be regarded as conservative, given that the incidence of type 2 diabetes is likely to increase in coming years [2].

### Supporting scenarios

Results from the supporting scenarios showed an increase in clinical as well as economic benefits for EIMC over time. The highest benefits were found with higher weight loss and lower HbA1c targets over all examined time horizons, with the majority of benefits achieved in the first decade of treatment (see tables provided in Supplementary material). The benefits of EIMC were clear even in the most conservative scenario of a HbA1c level of 6.9% and a weight loss of 1.5 kg with cost savings of EUR 1,107 and an increase in quality-adjusted life expectancy of 0.031 years after 10 years of treatment. These benefits increased with higher treatment targets and longer time horizons, showing that these two markers were the most important clinical drivers.

## Discussion

The present study is the first to quantify the potential benefits of EIMC compared with conventional metabolic control for patients with type 2 diabetes in Italy. Long-term projections with a validated model of type 2 diabetes showed that EIMC was associated with notable improvements in life expectancy and quality-adjusted life expectancy, as well as reductions in the direct and indirect costs of diabetes-related complications. At the population level for the 50-year estimate, EIMC was projected to add 33,112 years of life compared with conventional management, which is the equivalent of 55 additional days for each patient. The reduced risk of complications with EIMC led to even greater improvements in quality-adjusted life expectancy, with approximately 55,403 additional QALYs for the population diagnosed in 2021. The clinical benefits associated with EIMC also led to improved cost outcomes compared with conventional management. Over a lifetime horizon, the cost savings for complications avoided amounted to EUR 2,902 per patient and EUR 628.0 million for the population diagnosed in 2021. A large part of these benefits were already accrued after only 10-years of the simulation, in which nearly 80% of total cost savings were realized and almost 60% of the quality-adjusted life years were gained at the population level. Assuming that EIMC could be established as part of routine diabetes management in Italy, the economic burden of diabetes-related complications in newly diagnosed patients with type 2 diabetes could be reduced by approximately 32% over the next decade [35]. Scenarios investigating smaller improvements in HbA1c and lesser reductions in body weight were run to provide supporting evidence for EIMC, whether reaching gold standard targets or not, and quantify the clinical and cost benefits versus conventional management at a range of HbA1c and body weight targets (see *Supplementary Material*). These scenarios suggest that any form of EIMC, even those less stringent that reported here, represent a worthwhile investment of healthcare resources.

Evidence examining the effectiveness of EIMC supports a shift in the treatment paradigm for type 2 diabetes and suggests that the traditional approach of gradually intensifying treatment is not the most effective option for a certain part of the affected population, from a clinical as well as economic standpoint [11]. It is important to note in this regard that whilst the EIMC treatment approach modeled here is focused on more stringent HbA1c and body weight targets than conventional management, it remains in line with individual, patient-centered care conforming with published guidelines not only in Italy, but also in Europe and North America [4, 8]. The treatment paradigm for type 2 diabetes has shifted in recent years towards early, personalized intervention, gradually moving away from traditional treatment intensification steps in response to chronic hyperglycemia, which in the past has led to sub-optimal glycemic control and an increased risk of diabetes-related complications [8]. The present analysis is a first step to understanding the humanistic and economic impact of more intensive therapy, early in the disease progression. Moreover, the introduction of more and more therapies that are weight neutral or promote weight loss may represent an opportunity to target more stringent metabolic goals for patients with type 2 diabetes. These observations, allied to the present analysis quantifying the clinical and economic benefits of treating newly diagnosed patients with type 2 diabetes early on with aggressive clinical targets, suggests that it may be time to revisit the management paradigm for type 2 diabetes. Treatment targets should be set with EIMC in mind, to provide the most benefit for the individual patients from a clinical outcomes perspective. This approach is likely to lead to improved economic outcomes for the healthcare payer and a reduction in the economic burden associated with the disease.

A limitation of the present analysis is that it did not include the costs associated with therapeutic interventions for the management of type 2 diabetes, and it is reasonable to expect that economic savings associated with EIMC would be eroded by the costs of the treatments required to achieve glycemic and weight loss targets. EIMC would likely involve a range of antihyperglycemic medications including older and newer treatments such as metformin, SGLT-2 inhibitors and GLP-1 receptor agonists, as well as lifestyle and educational interventions. Given the unknown make-up of treatment regimens required to meet EIMC treatment targets and how they might change over time, estimating pharmacy costs would have been very challenging for the present analysis (and would have been a source of considerable uncertainty). Lifetime cost savings were approximately EUR 3,600 per patient (undiscounted) for patients who reach early glycemic control and weight loss targets, with a duration of benefit of 6 years (after which point treatment is assumed to be the same as in the conventional management arm). Using this additional budget for more intensive therapy for patients with newly diagnosed type 2 diabetes could improve clinical outcomes for the population at no increased cost to the payer.

As with any long-term health economic modeling study, the current analysis is associated with several limitations which are important to acknowledge to contextualize the findings. Any long-term modeling analysis is inherently associated with a degree of uncertainty due, most notably, to unknown factors such as future changes to the management of complications, disease demographics and healthcare funding. Whilst managing many of these potential uncertainties is intractable, effort was used to minimize uncertainty in the present modeling analysis by using a recently developed model of type 2 diabetes that has been published and validated against real-life clinical outcomes data [12]. Despite EIMC interventions applied in the model being hypothetical, the treatment effects applied in the analysis were aligned with those found in recent studies and can therefore be seen as achievable targets in modern clinical practice. This approach to EIMC was assumed to be representative of what might be possible in a newly diagnosed cohort with type 2 diabetes in Italy. Each patient will, of course, be different with some above or below the HbA1c target and some above or below the weight loss target but at the population level, approaching the targets used in the analysis of EIMC would be expected to produce corresponding benefits in life expectancy, quality-adjusted life expectancy, complication rates and costs in routine clinical practice. It should also be noted that the current study only looks at two risk factors. Weight loss is likely to be accompanied by improvements in blood pressure and serum lipid levels, which also contribute to the risk of diabetes-related complications. As a result, the outcomes reported for EIMC in the present analysis could be considered conservative and may be an underestimation of the actual benefits in routine clinical practice. It is also noteworthy that the present analysis only focused on conventional risk factors and did not take into account the potential cardioprotective benefits associated with many new treatments that have been reported in cardiovascular outcomes trials [36]. Factoring in additional cardioprotective benefits could make the long-term improvements in clinical outcomes associated with EIMC even greater than those reported here.

This is the first study to quantify the clinical and economic benefits of a treatment paradigm shift to EIMC in the management of type 2 diabetes. The magnitude of benefits associated with EIMC in the Italian setting are likely to be reflected in other Western countries, where the healthcare systems are broadly analogous. EIMC will likely be associated with improved clinical outcomes for newly diagnosed patients with type 2 diabetes, although the magnitude of those benefits and the associated cost savings may change between countries. Recent expansion of the therapeutic options for the management of type 2 diabetes to include treatments that can improve glycemic control without increasing body weight have created new opportunities to improve the standard of care. In line with previously published studies that showed that early improvements in risk factors can lead to benefits in long-term outcomes, the present analysis quantifies the potential benefits of EIMC in an Italian setting [10, 11]. This modeling study suggests that achieving strict HbA1c targets and clinically significant weight loss within a year from diagnosis may reduce the economic burden and improve health outcomes for patients living with type 2 diabetes in Italy.

## Supplementary information

Below is the link to the electronic supplementary material.

ESM 1 (DOCX 702 KB)

## Data Availability

The study used data previously collected and published in an aggregated form. All used data is shown in the main manuscript and in provided in the Supplementary material.
