# Supplementary material for: Improving outcomes with early and intensive metabolic control in patients with type 2 diabetes: a long-term modeling analysis of clinical and cost outcomes in Italy
Source: J Diabetes Metab Disord. 2025 Jan 29;24(1):58. doi: 10.1007/s40200-024-01553-w (PMC11780239; doi:10.1007/s40200-024-01553-w)
Supplement: Supplementary file 1 — (DOCX 702 KB) [file 40200_2024_1553_MOESM1_ESM.docx]

Improving outcomes with early and intensive metabolic control in patients with type 2 diabetes: a long-term modeling analysis of clinical and cost outcomes in Italy

Journal of diabetes and Metabolic Disorders

Pierluca Arietti^1^, Kristina Secnik Boye^2^, Maurizio Guidi^1^, Jonathan Rachman^3^, Marco Orsini Federici^1^, Rosanna Raiola^1^, Arianna Avitabile^1^, William Valentine^4^

^1^ Eli Lilly and Company, Sesto Fiorentino, Italy

^2^ Eli Lilly and Company, Indianapolis, IN, USA

^3^ Eli Lilly and Company, Basingview, Hampshire, UK

^4^ Ossian Health Economics and Communications GmbH, Basel, Switzerland

valentine@ossianconsulting.com

10-year time horizon results

Table 1 Potential cost savings per patient with EIMC versus conventional metabolic control in Italy over the next 10 years for patients newly diagnosed with type 2 diabetes

|  | | **Target HbA1c (%)** | | | | |
| --- | --- | --- | --- | --- | --- | --- |
|  |  | **5.7%** | **6.0%** | **6.3%** | **6.6%** | **6.9%** |
| **Target weight loss (kg)** | **1.5** | 2,001 | 1,816 | 1,524 | 1,227 | 1,107 |
|  | **3.5** | 2,027 | 1,951 | 1.589 | 1,420 | 1,144 |
|  | **5.5** | 2,269 | 2,014 | 1,870 | 1,608 | 1,332 |
|  | **7.5** | 2,353 | 2,104 | 1,875 | 1,623 | 1,500 |
|  | **9.5** | 2,550 | 2,281 | 2,026 | 1,816 | 1,523 |

HbA1c, glycated hemoglobin

Table 2 Potential (discounted) quality-adjusted life expectancy improvements per patient with EIMC versus conventional metabolic control in Italy over the next 10 years for patients newly diagnosed with type 2 diabetes

|  | | **Target HbA1c (%)** | | | | |
| --- | --- | --- | --- | --- | --- | --- |
|  |  | **5.7%** | **6.0%** | **6.3%** | **6.6%** | **6.9%** |
| **Target weight loss (kg)** | **1.5** | 0.073 | 0.062 | 0.057 | 0.043 | 0.031 |
|  | **3.5** | 0.095 | 0.086 | 0.078 | 0.068 | 0.054 |
|  | **5.5** | 0.119 | 0.113 | 0.097 | 0.089 | 0.077 |
|  | **7.5** | 0.142 | 0.131 | 0.121 | 0.113 | 0.099 |
|  | **9.5** | 0.163 | 0.152 | 0.145 | 0.133 | 0.121 |

HbA1c, glycated hemoglobin

20-year time horizon results

Table 3 Potential cost savings per patient with EIMC versus conventional metabolic control in Italy over the next 20 years for patients newly diagnosed with type 2 diabetes

|  | | **Target HbA1c (%)** | | | | |
| --- | --- | --- | --- | --- | --- | --- |
|  |  | **5.7%** | **6.0%** | **6.3%** | **6.6%** | **6.9%** |
| **Target weight loss (kg)** | **1.5** | 2,213 | 2,012 | 1,630 | 1,279 | 1,275 |
|  | **3.5** | 2,305 | 2,315 | 1,792 | 1,667 | 1,276 |
|  | **5.5** | 2,626 | 2,313 | 2,309 | 1,872 | 1,563 |
|  | **7.5** | 2,814 | 2,513 | 2,324 | 1,948 | 1,905 |
|  | **9.5** | 3,203 | 2,526 | 2,515 | 2,369 | 1,892 |

HbA1c, glycated hemoglobin

Table 4 Potential (discounted) quality-adjusted life expectancy improvements per patient with EIMC versus conventional metabolic control in Italy over the next 20 years for patients newly diagnosed with type 2 diabetes

|  | | **Target HbA1c (%)** | | | | |
| --- | --- | --- | --- | --- | --- | --- |
|  |  | **5.7%** | **6.0%** | **6.3%** | **6.6%** | **6.9%** |
| **Target weight loss (kg)** | **1.5** | 0.154 | 0.132 | 0.115 | 0.093 | 0.067 |
|  | **3.5** | 0.180 | 0.156 | 0.143 | 0.116 | 0.090 |
|  | **5.5** | 0.200 | 0.188 | 0.158 | 0.144 | 0.120 |
|  | **7.5** | 0.230 | 0.206 | 0.185 | 0.168 | 0.142 |
|  | **9.5** | 0.249 | 0.259 | 0.212 | 0.189 | 0.169 |

HbA1c, glycated hemoglobin

50-year time horizon results

Table 5 Potential cost savings per patient with EIMC versus conventional metabolic control in Italy over the next 50 years for patients newly diagnosed with type 2 diabetes

|  | | **Target HbA1c (%)** | | | | |
| --- | --- | --- | --- | --- | --- | --- |
|  |  | **5.7%** | **6.0%** | **6.3%** | **6.6%** | **6.9%** |
| **Target weight loss (kg)** | **1.5** | 2,265 | 1,916 | 1,520 | 1,188 | 1,252 |
|  | **3.5** | 2,139 | 2,315 | 1,733 | 1,629 | 1,226 |
|  | **5.5** | 2,616 | 2,283 | 2,322 | 1,855 | 1,539 |
|  | **7.5** | 2,819 | 2,567 | 2,383 | 1,969 | 1,942 |
|  | **9.5** | 3,273 | 2,902 | 2,571 | 2,478 | 1,953 |

HbA1c, glycated hemoglobin

Table 6 Potential (discounted) quality-adjusted life expectancy improvements per patient with EIMC versus conventional metabolic control in Italy over the next 50 years for patients newly diagnosed with type 2 diabetes

|  | | **Target HbA1c (%)** | | | | |
| --- | --- | --- | --- | --- | --- | --- |
|  |  | **5.7%** | **6.0%** | **6.3%** | **6.6%** | **6.9%** |
| **Target weight loss (kg)** | **1.5** | 0.180 | 0.154 | 0.133 | 0.105 | 0.076 |
|  | **3.5** | 0.211 | 0.179 | 0.170 | 0.130 | 0.100 |
|  | **5.5** | 0.232 | 0.209 | 0.181 | 0.167 | 0.135 |
|  | **7.5** | 0.265 | 0.236 | 0.215 | 0.187 | 0.161 |
|  | **9.5** | 0.286 | 0.256 | 0.243 | 0.213 | 0.191 |

HbA1c, glycated hemoglobin
